# High-Selective CO_2_ Capture in Amine-Decorated Al-MOFs

**DOI:** 10.3390/nano12224056

**Published:** 2022-11-17

**Authors:** Yinji Wan, Yefan Miao, Ruiqin Zhong, Ruqiang Zou

**Affiliations:** 1 State Key Laboratory of Heavy Oil Processing, China University of Petroleum, No. 18 Fuxue Road, Changping District, Beijing 102249, China; 2 Beijing Key Laboratory for Theory and Technology of Advanced Battery Materials, School of Materials Science and Engineering, Peking University, No. 5 Yiheyuan Road, Haidian District, Beijing 100871, China

**Keywords:** MOF-520, amine-functionalized MOF-520, CO_2_ capture and separation, CO_2_/N_2_ selectivity

## Abstract

Amine-functionalized metal-organic framework (MOF) material is a promising CO_2_ captor in the post-combustion capture process owing to its large CO_2_ working capacity as well as high CO_2_ selectivity and easy regeneration. In this study, an ethylenediamine (ED)-decorated Al-based MOFs (named ED@MOF-520) with a high specific area and permanent porosity are prepared and evaluated to study the adsorption and separation of CO_2_ from N_2_. The results show that ED@MOF-520 adsorbent displays a superior CO_2_ capture performance with a CO_2_/N_2_ separation factor of 50 at 273 K, 185% times increase in the CO_2_/N_2_ separation efficiency in comparison with blank MOF-520. Furthermore, ED@MOF-520 exhibits a moderate-strength interaction with 29 kJ mol^−1^ adsorption heat for CO_2_ uptake, which not only meets the requirement of CO_2_ adsorption but also has good cycle stability. This work provides a promising adsorbent with a high CO_2_/N_2_ separation factor to deal with carbon peak and carbon neutrality.

## 1. Introduction

Carbon capture and storage/sequestration (CCS) has been proposed as a commercially viable technology to abate CO_2_ emissions in the post-combustion capture process [1,2,3]. The core part of CCS is the development of highly efficient CO_2_ captors. Therefore, numerous research efforts have been devoted to the following aspects such as chemical absorption by amine solutions [4,5,6,7], the cryogenic separation distillation method [8], the membrane separation method [9,10,11,12], and the adsorption method using porous solid adsorbents [13,14,15,16,17,18,19,20,21,22,23,24]. It is widely believed that the adsorption and separation of CO_2_ by solid adsorbents has enormous potential such as activated carbon, Li-based ceramics, and MOFs, etc. due to environmental benignity, economic cost, and flexible operability [25,26].

Among them, metal-organic frameworks (MOFs), constructed by metal ions/clusters and organic linkers by coordination bonds are considered to be the most competitive candidates for CO_2_ capture and separation owing to their high surface area and tunable pore sizes and chemical environments of pore [27,28,29,30,31,32]. Unfortunately, CO_2_ is often physically adsorbed in channels of MOFs, thus leading to the low selectivity of CO_2_ capture in coexisting species (N_2_, CO, and H_2_O) [33,34,35]. One of the efficient solutions is to fabricate amine-functionalized MOFs materials with uncoordinated and electron-rich N atoms, which can act as the active adsorption sites of CO_2_ through Lewis acid−base chemistry between CO_2_ and amine, thereby realizing high-selectivity CO_2_ adsorption and separation [36,37,38]. The introduction of amine molecules into MOFs has been prepared by pre- and post-synthetic modification strategies [39,40,41]. For example, Zhong et al. [39] introduced tris(2-aminoethyl) amine (TAEA), ethylenediamine (ED), and triethylenediamine (TEDA) into open Cr sites of MIL-101(Cr) by the double-solvent method, respectively. The authors studied the effect of amine species grafted-MIL-101(Cr) on the adsorption and separation performance of CO_2_/CO. The experimental and theoretical results showed that the CO_2_/CO selectivity of TAEA@MIL-101was 103 times higher than that of unmodified MIL-101. In another work, a diamine-grafted into the ligands of IRMOF-74-III was designed and developed by Yaghi and coworkers for CO_2_ capture in the presence/absence of H_2_O, providing fundamental insights of CO_2_ adsorption in the framework chemistry of MOFs [42]. Mason et al. [43] found that amine-appended Mg_2_(dobpdc) displays an outstanding CO_2_ adsorption capacity with up to 4.2 mmol g^−1^ under high H_2_O partial pressure. McDonald et al. [44] further unveiled the adsorption mechanism of these amine-modified MOFs, firstly, the H transfer from the -NH_2_ and the nucleophilic attack of N on CO_2_ leads to the formation of ammonium carbamate species, which destabilizes the amine at the next metal site, thus inducing CO_2_ synergistic adsorption via chain reactions. Wan et al. [41] used a mixed ligands strategy to synthesize a series of amine-grafted Ti-MOFs (MIP-207-NH_2_) for trapping CO_2_ from N_2_, they found that the performance of CO_2_ capture of MIP-207-NH_2_ functions as the contents of the amino group. Among these adsorbents, the MIP-207-NH_2_-25% possessed a 3.96 mmol g^−1^ CO_2_ capture capacity with the highest CO_2_/N_2_ separation factor (77) at 273 K, which was about 21% and 33% higher than those of pure MIP-207. However, novel CO_2_ adsorbents with high selectivity and large CO_2_ adsorption capacity need to be further developed and explored. Up to now, there have not been studies on MOF-520 [Al_8_(μ-OH)_8_(HCOO)_4_(BTB)_4_, BTB = 1,3,5-benzenetribenzoate] for CO_2_ uptake and amine-modified MOF-520 is not reported yet.

MOF-520 was chosen as the porous material mainly considering the high specific surface area with large micropores (16.2 × 9.9 Å) [45], which could offer adequate space for the introduction of organic amine molecules and confine them in the channel via van der Waals force considering MOF-520 does not have strong binding sites (i.e., open metal sites). In addition, the introduction of organic amine can regulate the pore size of materials and improve the separation effect of molecular sieves to a certain extent, therefore, facilitating the selective adsorption of CO_2._ In this study, the MOF-520 and ED-decorated MOF-520 (ED@MOF-520) were prepared, characterized and evaluated to study the adsorption and separation of CO_2_ from N_2_. The features of MOF-520 can uptake mixed gas CO_2_/N_2_ according to the different molecule kinetic diameters. After incorporating ED into the channels of MOF-520 via a double solvent approach, the ED@MOF-520 composites displayed a superior CO_2_ capture performance with a CO_2_/N_2_ separation factor of 50, 185% times higher than that of unmodified MOF-520 at 273 K. The high selective enhancement mechanism relied on the introduction of electron donor amine which can preferentially trap CO_2_ from N_2_ through Lewis acid−base interactions between CO_2_ and amine.

## 2. Experimental Section

### 2.1. Synthesis of MOF-520

All the reagents were purchased from commercial sources and were used without further purification. MOF-520 was synthesized by hydrothermal reaction of Al(NO_3_)_3_·9H_2_O (90 mg), BTB (75 mg), deionized water (40 μL), DMF (13 mL), formic acid (1.9 mL), and anhydrous ethanol (24 mL) at 140 °C for 14 h according to the previous procedure [45]. After that, the solid was separated and washed with anhydrous acetone. Finally, the products were obtained by vacuum-drying at 120 °C for 8 h.

### 2.2. Synthesis of ED@MOF-520

**Figure 1:**
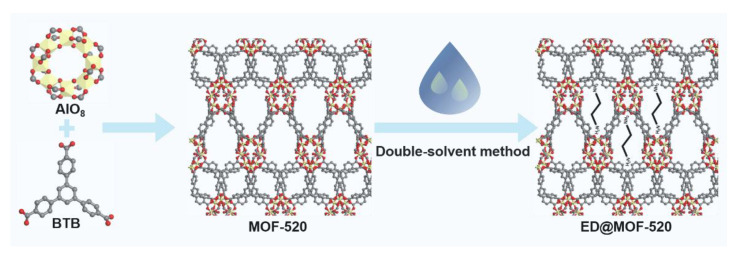
Synthesis schematic diagram of MOF-520 and ED@ MOF-520, where the yellow, red, and gray color represents Al, O, and C atom, respectively.

The ED@MOF-520 was prepared by squeezing ED into the pores of activated MOF-520 (Figure 1). Firstly, the synthesized MOF-520 was activated at 120 °C for 8 h in a vacuum oven. The activated MOF-520 (150 mg) was added to n-hexane (20 mL) and dispersed by ultrasonic treatment for 20 min. Subsequently, 32 μL ED (the loading amount of ED accounts for 20% of the pore volume of MOF-520) was gradually added into the above-mentioned solution using the syringe pump under vigorous stirring. After that, the mixture was sealed and stirred for 4 h. Then, the vial was opened and stirred until the n-hexane was fully evaporated. Finally, the product was activated under vacuum conditions at 120 °C for 8 h to remove residual n-hexane and the external surface ED, and a yellow solid product was denoted as ED@MOF-520-20%.

### 2.3. Sample Characterization and Gas Adsorption Measurements

Powder X-ray diffraction (PXRD) patterns for the samples were performed on a Rigaku D/max 2400 X-ray diffractometer equipped with Cu Kα radiation (λ = 1.5406 Å). Thermogravimetric analyzes (TGA) were performed on an SDT Q600 analyzer (New Castle, DE, USA). The test of adsorption and desorption cyclic stability was carried out on SDT Q600analyzer. C, H, and N contents of samples were measured by elemental analysis (Elementar Vario EL CUBE (Langenselbold, Germany). Fourier-transform infrared spectroscopy (FTIR) spectra of all samples were collected by a Bruker Tensor 27 Fourier transform infrared spectrometer. Nitrogen adsorption isotherms at 77 K were performed within the pressure range 0–1 atm (Quantachrom Autosorb-IQ gas adsorption analyzer). The specific surface area was calculated with the Brunauer-Emmett-Teller (BET) model and pore size distribution was obtained by the non-local density functional theory (NLDFT) model. The N_2_ at 77 K on carbon (slit pore, NLDFT equilibrium model) was selected as the calculation model to obtain the pore size distribution profile. The details about the CO_2_ and N_2_ adsorption measurements can be found in our previous report [41].

## 3. Results and Discussion

### 3.1. Structural Analysis of Sample

**Figure 2:**
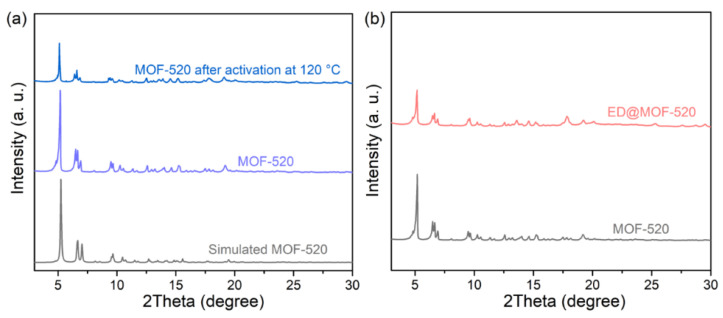
(**a**) PXRD patterns of MOF-520, MOF-520 after activation at 120 °C and simulated one from crystal structure data of MOF-520, (**b**) PXRD patterns of MOF-520 and all ED@MOF-520.

As shown in Figure 2a, the characteristic diffraction peaks of MOF-520 at 2*θ* = 5°, 6.5°, and 7° can be observed after hydrothermal treatment at 140 °C for 14 h [45]. Compared with simulated MOF-520, the peak position and relative intensities are basically the same, illustrating that MOF-520 was successfully synthesized. It should be pointed out that the peak splitting at 6.5° may be associated with the presence of solvent molecules incorporating the micropores during the MOF-520 synthesis [46]. Figure 2a also exhibits that the structure of MOF-520 maintains well after the thermal activation temperature of 120 °C. From Figure 2b, the amount of loading ED has a great effect on the crystal structure of MOF-520, where the peak relative intensities of ED@MOF-520 gradually decrease with the amount of ED increasing, indicating that the crystallinity of MOF-520 gradually decreases. The crystal structure of MOF-520 is maintained in the ED@MOF-520 composites when the amount of ED added less than 30% (Figure S1). However, the main peaks of MOF-520 have completely disappeared when the amount of ED increases to 40%, and no obvious peaks can be observed in the ED@MOF-520-60% (Figure S1). Our findings lead us to conclude that the functional groups (–COOH) of ligands of MOF-520 can react with the -NH_2_ groups of ED, and the framework of MOF-520 can be damaged with the amount of ED over 40%.

**Figure 3:**
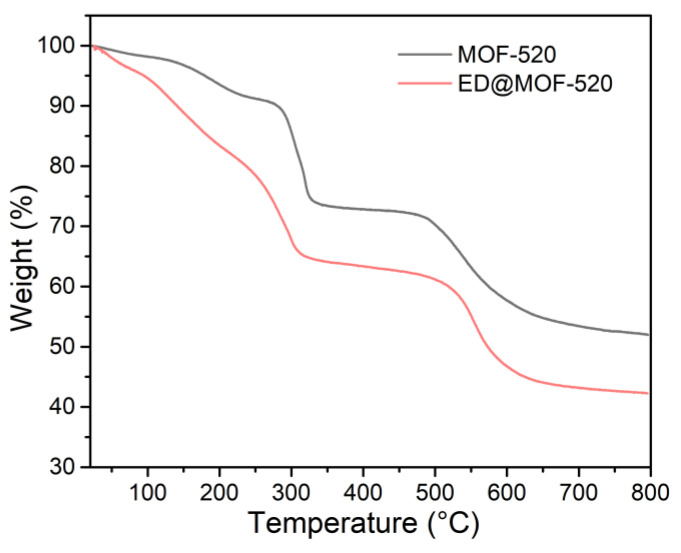
TGA curves of samples.

TGA technology was used to trace the effect of ED on the thermal stability of the materials. As shown in Figure 3, the ED@MOF-520 composite showed a similar TGA curve trend as the pristine MOF-520, containing three-step weight loss, which was attributed to the removal of guest molecules (ED and solvents used), decomposition of organic ligand, and collapse of parent frameworks [39]. It can be seen that the difference in TGA curves of ED@MOF-520 and MOF-520 is mainly manifested in the first stage of weight loss (<230 °C). Compared with MOF-520 (~74.10%), the ED@MOF-520 lost weight to about 65.5% in the first step.

**Figure 4:**
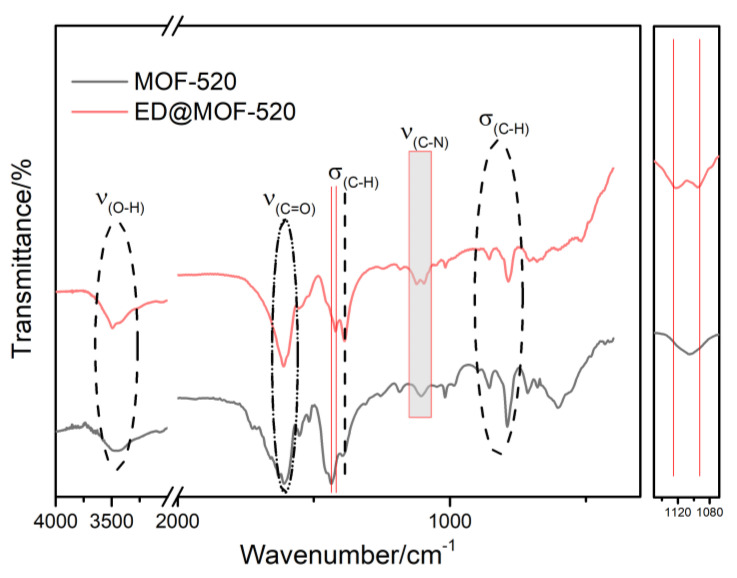
FT-IR results of samples.

To figure out the changes in the functional groups and chemical bonds of modified MOF-520, FT-IR tests were performed on MOF-520 and ED@MOF-520 (Figure 4). Prior to the FT-IR measurements, all samples were treated by vacuum drying at 120 °C for 8 h to remove the H_2_O and surface ED molecules of materials. For MOF-520, the strong adsorption peak at 3462 cm^−1^ is attributed to the stretching vibration peak of −OH, and the characteristic peak at 1608 cm^−1^ is clearly observed, which is attributed to the stretching vibration of the C=O bond of carboxyl. The peak at 1552~1430 cm^−1^ is the characteristic peak of the benzene ring, and the peak at 1388 cm^−1^ is found owing to the existence of bending vibration of the C−H bond. The peaks at 850 and 790 cm^−1^ can be to the out-of-plane deformation vibration characteristic peaks of the C–H bond on the benzene ring of the ligand. Compared with MOF-520, ED-modified MOF-520 materials maintain the original characteristic peaks of MOF-520. Notably, a −C−N peak was found at 1125 cm^−1^ by magnification, and the peak intensities of C−H at 1388 cm^−1^ were strengthened, indicating that ED molecules were successfully squeezed into the MOF-520 channels. In addition, there was a red-shifted phenomenon about the peak at 1436 cm^−1^ of MOF-520, moving to 1418 cm^−1^. The possible reason was that a hydrogen bond was formed between the N atom in ED and the H atom on the benzene ring. As a result, the C−H of the benzene ring shifted to a lower wavenumber, which also confirmed the introduction of ED into the MOF-520 pore. To quantitatively analyze the percentages of C, H, and N elements, the ED@MOF-520 material elemental analysis was performed. As shown in Table 1, the N element was not detected in the parent MOF-520. With the addition of ED content, the content of both N and H elements increased, further indicating that ED has been appended to the framework of MOF-520. It should be pointed out that the loading content of N obtained by elemental analysis was less than the difference in weight loss between MOF-520 and ED@MOF-520, which may be associated with more solvent molecules.

**Figure 5:**
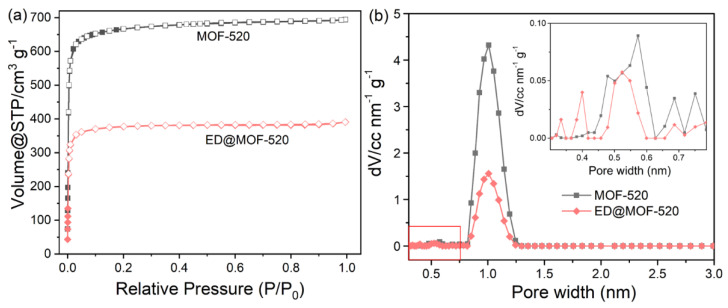
(**a**) N_2_ adsorption and desorption isotherms, (**b**) pore size distribution curves (Inset: pore distribution curves in the interval of 0 to 0.8 nm).

The results of N_2_ physisorption isotherms measurements of samples are shown in Figure 5a and Table 1. According to the IUPAC classification, the N_2_ adsorption-desorption curves of materials belong to I-type with no hysteresis loops related to microporous characteristics. Additionally, the specific surface areas are determined by N_2_ adsorption curves using the Brunauer-Emmett-Teller (BET) model. Table 1 shows that the BET area of MOF-520 is 2699.46 m^2^ g^−1^ and BET areas of ED@MOF-520 decreased with the increase in ED loading. Based on the above results and PXRD analysis, the polar ED molecules have been successfully squeezed and retained into the pore of MOF-520 by the capillary enrichment effect and the van der Waals force. To accurately assess the influence of ED on the pore size of the material, the carbonaceous slit model of the NLDFT method was used to obtain the pore size distribution of both MOF-520 and ED@MOF-520 composites (Figure 5b). It can be seen that the pore diameter of 1.007 nm of MOF-520 gradually declines with ED loaded, and the two kinds of micropore with a pore diameter of 0.686 and 0.75 nm disappear after ED modification. These phenomena fully indicate the ED preferentially fills into the relatively large pores of MOF-520. Meanwhile, the pore size of ED@MOF-520 has moved from 0.57 to 0.52 nm, and a smaller pore size of less than 0.4 nm is produced.

### 3.2. Gas Adsorption Performance of Adsorbents

**Figure 6:**
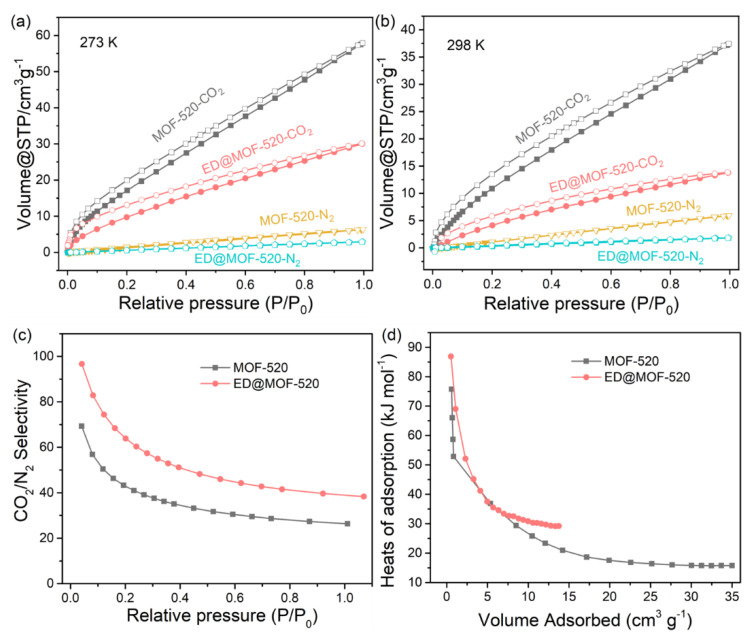
CO_2_ and N_2_ adsorption and desorption isotherms of ED@MOF-520 materials with different ED content at (**a**) 273 K and (**b**) 298 K, (**c**) CO_2_/N_2_ selectivity at 273 K, (**d**) CO_2_ adsorption enthalpy curves.

The CO_2_ and N_2_ adsorption performance at 273 and 298 K are shown in Figure 6a,b and Table 2. Apparently, The CO_2_ uptakes of ED@MOF-520 are lower than that of MOF-520 at 273 and 298 K, respectively. Combined with the pore sizes curve (Figure 6b), it can be seen that MOF-520 possesses an abundant microporous structure, hence behaving with an outstanding CO_2_ capture capacity. Although MOF-520 after ED grafting can increase the number of adsorption sites, the overall CO_2_ uptakes of ED-modified MOF-520 composites reduce, which is caused by the sharp decrease in the BET area (Table 1). With the addition of ED, the pores where CO_2_ molecules diffuse (pores with a pore diameter larger than the CO_2_ molecular dynamics diameter of 0.33 nm) gradually decrease. Therefore, the amount of CO_2_ absorbed by the material is affected by both the number of adsorption sites and the pore sizes.

In order to further study the adsorption separation performance of materials for CO_2_/N_2_, the typical Ideal Adsorption Solution Theory (IAST) method was used to calculate the separation factor of CO_2_/N_2_ of MOF-520 and ED@MOF-520 series materials at 273 K (Figure 6c and Figure S2c) [50]. It can be found that the separation performance of ED@MOF-520 is the best under 1 atm, and the separation factor reaches 50, which is nearly 50% higher than the separation factor (27) of MOF-520. There may be two reasons for the higher selectivity of ED@MOF-520. (i) the presence of amine groups increases the interaction between the ED@MOF-520 composite and CO_2_ molecules; (ii) as the pore size decreases in ED@MOF-520, there are smaller pores at 3.3, 4, and 5.2 Å, and the molecular sieving effect and confinement effect are enhanced [51,52], thereby promoting the selective adsorption of CO_2_. Besides, the adsorption separation performance of CO_2_ of ED@MOF-520-10% and 30% composites were further analyzed (supporting information), indicating that ED@MOF-520 adsorbents display a better CO_2_ capture performance. Additionally, ED@MOF-520 displays good adsorption and separation of CO_2_ performance among these reported amine-modified MOFs adsorbents (Table 2).

To quantitatively understand the CO_2_-MOF interactions, the isosteric heats of adsorption (Q_st_) (calculation details can be found in the supporting information) were calculated from the CO_2_ adsorption isotherms at 273 and 298 K (Figure 6d and Figure S2d). Initially, the Q_st_ of ED@MOF-520 for CO_2_ is as high as 90 kJ mol^−1^, which is higher than that of pristine MOF-520 (75 kJ mol^−1^). It can be attributed to the strong bonding interaction between CO_2_ and the Lewis basic sites (i.e., uncoordinated and electron-rich N atoms) and the hydrogen bond of the ED@MOF-520 framework. With the increase in CO_2_ uptake, the Q_st_ of CO_2_ by the adsorbents gradually decreases and tends to be stable. Finally, the Q_st_ of ED@MOF-520 is stable at 29 kJ mol^−1^, indicating a moderate-strength interaction with CO_2_ [38].

**Figure 7:**
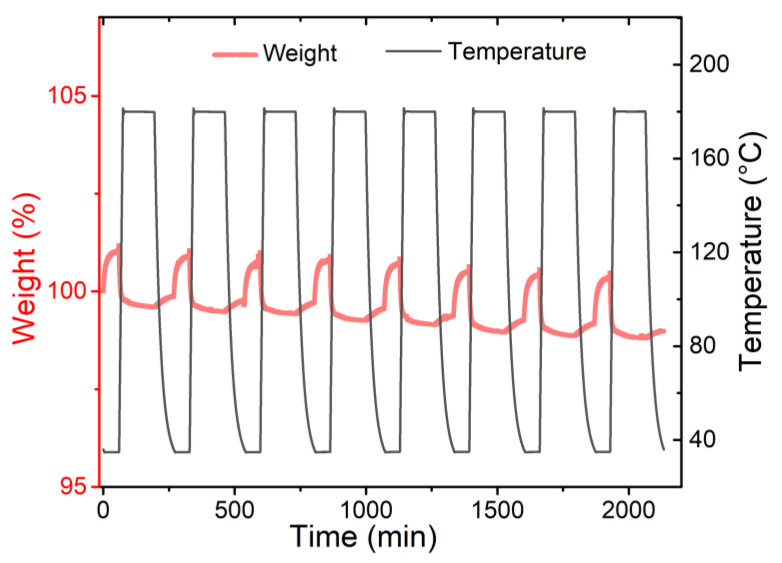
CO_2_ adsorption and desorption cycle curves of ED@MOF-520.

The thermogravimetric analyzer was used to investigate the stability of CO_2_ adsorption-desorption of ED@MOF-520, First of all, the sample was kept at 35 °C for 1 h to fully adsorb CO_2_, and then it switched to N_2_ purge and raised the temperature to 180 °C for 2 h to desorb CO_2_ from the sample. As shown in Figure 7, the CO_2_ uptake of ED@MOF-520 in the eight cycles is not significantly reduced, which reveals good adsorption and desorption cycle stability.

## 4. Conclusions

In summary, the ED@MOF-520 composite was successfully synthesized by a double solvent method. The PXRD, FT-IR, and elemental analysis indicate the ED was deposited and confined onto the channels of MOF-520 physically. Although the incorporation of ED into MOF-520 could reduce the CO_2_ capture capacity, the generation of new adsorption sites of ED@MOF-520 leads to an improvement in the CO_2_/N_2_ separation performance. Additionally, the BET analysis displayed that the introduction of ED into host MOF-520 could induce a smaller pore size, which could enhance the molecular sieving effect of ED@MOF-520 and promote CO_2_ capture. This work confirms that the CO_2_ adsorption and separation performance is the result of the combination of adsorption sites and the BET area as well as the pore sizes of adsorbents.

## Figures and Tables

**Table 1:** The summary of BET data and elements content of C, H, and N of samples.

Sample	Surface Area (m^2^ g^−1^)	Pore Volume (cm^3^ g^−1^)	The Elements Content of C, H, and N (wt%)		
			C	H	N
MOF-520	2699.46	1.08	56.12	3.34	0
ED@MOF-520	787.44	0.39	45.53	4.12	3.39

**Table 2:** The comparison of BET area and CO_2_ capture performance in this work and reported amine-functionalized MOFs.

Samples	BET Area (m^2^ g^−1^)	CO_2_ Capture Capacity @ Testing Conditions	CO_2_/N_2_ (CO) Selectivity	Q_st_ (kJ mol^−1^)	Ref
MOF-520	2699.46	2.58/1.67 mmol g^−1^ @ 273/298 K &1 bar	27		This work
ED@MOF-520	787.44	1.55/0.61 mmol g^−1^ @ 273/298 K &1 bar	50	29	This work
ZrBDC-NH_2_-1:1-0.2	930–1040	4.20/2.68 mmol g^−1^ @ 273/298 K &1 bar	71.6	26.9	[47]
MIP-207	563	3.28/2.03 mmol g^−1^@ 273/298 K &1 bar	59	–	[41]
MIP-207-NH_2_ -25%	735	3.96/2.91 mmol g^−1^@ 273/298 K &1 bar	77	30–35	[41]
ED@Cu_3_(BTC)_2_-1	444	4.28/2.15 mmol g^−1^ @ 273/298 K &1 bar	21.5	39	[40]
ED@Cu_3_ (BTC)_2_-2	163	1.03/0.54 mmol g^−1^ @ 273/298 K &1 bar	2.68	–	[40]
ED@MIL-101	1584.6	3.93/1.93 mmol g^−1^ @ 273/298 K &1 bar	17.3	–	[39]
MIL-101(Cr)-NH_2_	~2800	3.4 mmol g^−1^ @ 289 K and 1 bar	26.5	54.6	[48]
NH_2_-MIL-53	–	~1.5/~1.1 mmol g^−1^@ 273/298 K &1 bar	–	~56	[49]

## Data Availability

The data presented in this study are available on request from the corresponding author.

## Supplementary Materials

The following supporting information can be downloaded at: https://www.mdpi.com/article/10.3390/nano12224056/s1, Figure S1. PXRD patterns of MOF-520 and ED@MOF-520 with different ED loaded samples, Figure S2. CO_2_ and N_2_ adsorption and desorption isotherms of ED@MOF-520-10% and ED@MOF-520-30% at (a) 273 K and (b) 298K, (c) CO_2_/ N_2_ selectivity at 273 K, (d) CO_2_ adsorption enthalpy curves, Table S1. The summary of BET data and elements content of C, H, and N of ED@MOF-520-10% and ED@MOF-520-30%.
