# Supplementary material for: High-Selective CO2 Capture in Amine-Decorated Al-MOFs
Source: Nanomaterials (Basel). 2022 Nov 17;12(22):4056. doi: 10.3390/nano12224056 (PMC9697124; doi:10.3390/nano12224056)
Supplement: Supplementary file 1 [file nanomaterials-12-04056-s001.zip › nanomaterials-1992764-supplementary.pdf]

nanomaterials

MDPI

Supplementary Materials

High-Selective CO_2 Capture in Amine-Decorated Al-MOFs

Yinji Wan [1,†], Yefan Miao [1,†], Ruiqin Zhong [1,*] and Ruqiang Zou [2,*]

1 A State Key Laboratory of Heavy Oil Processing, China University of Petroleum, Beijing, No. 18 Fuxue Road, Changping District, Beijing 102249, China
2 Beijing Key Laboratory for Theory and Technology of Advanced Battery Materials, School of Materials Science and Engineering, Peking University, No. 5 Yiheyuan Road, Haidian District, Beijing 100871, China
* Correspondence: zhong2004@foxmail.com (R.Z.); rzou@pku.edu.cn (R.Z.)
† These authors contributed equally to this work.

The calculation of isosteric heat of adsorption
According to Claussius-Clapeyron equation:

$$\Delta H = (H1 - H2) = RT^2\left(\frac{\partial \ln P}{\partial T}\right)_\theta$$

$$Qst = qiso = \Delta H = (H1 - H2) = RT^2\left(\frac{\partial \ln P}{\partial T}\right)_\theta$$

$$\ln p = \left(-\frac{q_{is_0}}{RT} + C\right)_\theta$$

H1: Molar enthalpy of gas
H2: Partial molar enthalpy of the adsorption phase
P: Equilibrium absolute pressure (Torr)
T: Temperature (K)
θ: Coverage is defined as the adsorption capacity divided by the specific surface area of the adsorbent (V/V_m).
R: 0.00831KJ/mol k
Qst: the isosteric heat of adsorption (KJ/mol)

The heat of adsorption is determined by ln (*P*) vs. 1/*T*), and the slope of each isosteric line can be used to calculate the heat of adsorption at a specific adsorption amount under partial pressure of adsorbent in adsorption equilibrium system at different adsorption temperatures.

The amount of loaded ED is determined by the pore volume of MOF-520 and the density of ED. For the synthesis of ED@MOF-520-n (n=10%, 30%, 40%, 60%), the synthetic protocol was essentially the same as the procedure used for the typical synthesis of ED@MOF-520 sample, except for adding ED in the amount of 10%, 30%, 40%, 60% of the pore volume of MOF-520 instead of 20%, respectively.

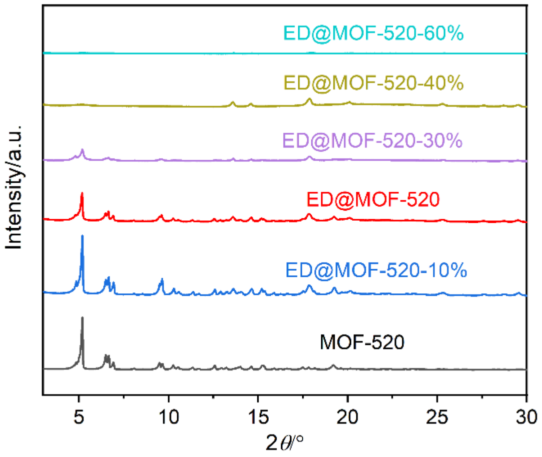

Figure S1. PXRD patterns of MOF-520 and ED@MOF-520 with different ED loaded samples.

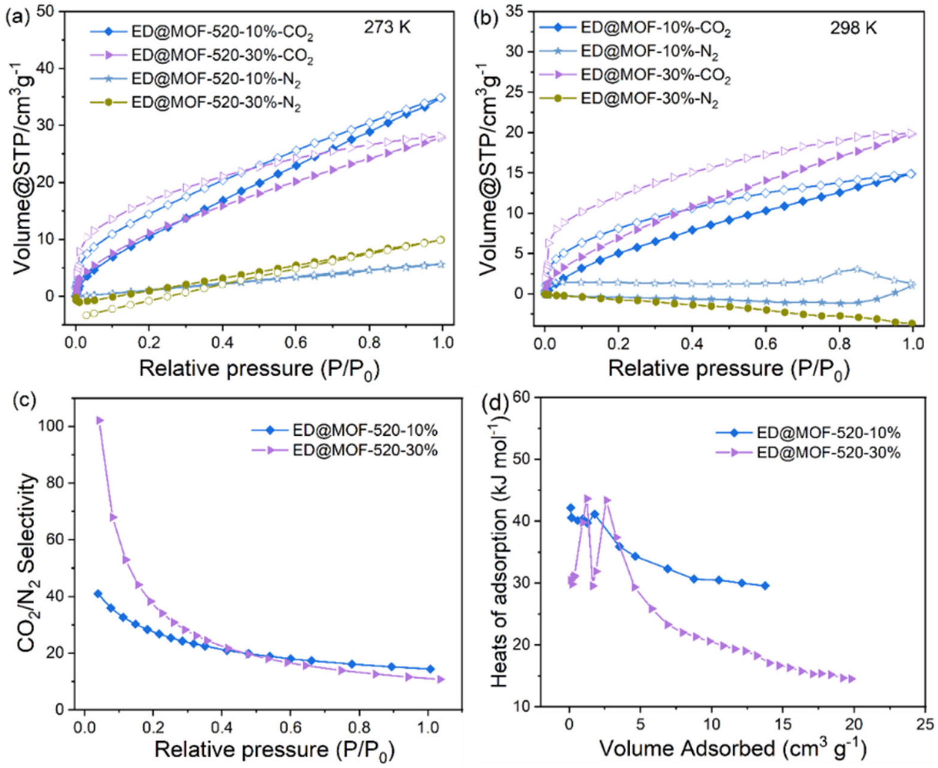

Figure S2. CO_2 and N_2 adsorption and desorption isotherms of ED@MOF-520-10% and ED@MOF-520-30% at (**a**) 273 K and (**b**) 298K, (**c**) CO_2/ N_2 selectivity at 273 K, (**d**) CO_2 adsorption enthalpy curves.

As the pressure increases, the CO_2 adsorption capacity of ED@MOF-520-10% is higher than that of ED@MOF-520 and ED@MOF-520-30%, demonstrating the influence of the reduced pore size on the adsorption capacity is stronger than the increase of adsorption sites number. At 298 K, the CO_2 capture capacity of ED@MOF-520-30% is higher than that of ED@MOF-520-10% and ED@MOF-520. In comparison with the BET results, (Fig. S2a-b), we can see that the specific surface area and pore volume of ED@MOF-520-30% are much lower than the other two ED-modified materials, which indicates that the numbers of adsorption sites play a dominant role in the adsorption process. In addition, the CO_2 adsorption and desorption curve of ED@MOF-520-30% exists a clear hysteresis loop,

showing that the presence of strong interaction between amine groups of the material and CO_2. The N_2 adsorption capacity is significantly lower than that of MOF-520, which fully manifests that the increase in the number of adsorption sites at 298 K has a greater impact on the adsorption performance than the reduction of pore size.

Table S1. The summary of BET data and elements content of C, H, and N of ED@MOF-520-10% and ED@MOF-520-30%.

Sample	Surface area (m^2 g^{-1})	Pore volume (cm^3 g^{-1})	The elements content of C, H, and N (wt%)		
			C	H	N
ED@MOF-520-10%	1536.42	0.61	44.70	3.62	2.07
ED@MOF-520-30%	307.20	0.15	45.65	4.84	7.39
